# Psychological Distress, Academic Stress, and Burnout among Saudi Undergraduate Nursing Students

**DOI:** 10.3390/jcm13123357

**Published:** 2024-06-07

**Authors:** Shaherah Yousef Andargeery, Murad H. Taani, Rania Ali Alhalwani, Heba E. El-Gazar

**Affiliations:** 1 Nursing Management and Education Department, College of Nursing, Princess Nourah bint Abdulrahman University, P.O. Box 84428, Riyadh 11671, Saudi Arabia; raalhalwani@pnu.edu.sa; 2 School of Nursing, University of Wisconsin, Milwaukee 1921 East Hartford Avenue, Milwaukee, WI 53211, USA; mhtaani@uwm.edu; 3 Nursing Administration Department, Faculty of Nursing Port Said University, Port Fouad City 42512, Port Said Governorate, Egypt; heba.emad@nur.psu.edu.eg

**Keywords:** depression, anxiety, stress, academic stress, burnout, undergraduate nursing students

## Abstract

**Background**: There is limited evidence on the association between psychological distress, academic stress, and burnout among Saudi nursing students. Clarifying such an association is crucial to understanding the factors associated with psychological distress and developing interventions to prevent it. **Aim:** To explore the prevalence and association of psychological distress with academic stress and burnout among Saudi nursing students. **Methods**: A cross-sectional design was used, and 237 students participated from a nursing college in Riyadh, Saudi Arabia. Students’ demographics; the Depression, Anxiety, and Stress Scale; the Academic Stress Inventory; and the Maslach Burnout Inventory were used for data collection. **Results**: Most of the participants reported no-to-mild depression, anxiety, and stress. Stress related to studying in groups, time management, emotional exhaustion, depersonalization, and personal accomplishment were the significant predictors of psychological distress, explaining 52.1% of the variance. **Conclusions**: This study suggest implementing tailored mental health screenings and support services for nursing students, embedding mental health professionals in the program, and using telehealth or mobile apps for remote monitoring to ensure comprehensive care for nursing students. Future research should consider these predictors while designing strategies to decrease psychological distress among students.

## 1. Introduction

Psychological distress pertains to a wide range of emotional and psychological symptoms. It comprises feelings of anxiety, depression, stress, and other mental health issues, which have a significant negative impact on an individual’s well-being and quality of life and contribute to the burden of mental health issues [1]. The widespread nature of psychological distress has been a matter of concern within the healthcare education system in recent years, especially among nursing students. Previous research reported a high prevalence of psychological distress among nursing students, ranging between 34% and 71% across different countries [2,3,4].

Nursing education is known for its demanding nature and rigorous theoretical curriculum. Increased heavy workloads, time constraints, and intense clinical experiences contribute to high levels of stress among nursing students. Nursing students are witnessing patient suffering and ethical dilemmas, have self-doubt or fear in relation to making mistakes, and deal with life-and-death situations during their clinical rotations, which can further exacerbate the psychological burden [5,6,7]. The coupled pressure to excel academically with the responsibility of providing competent and compassionate care to patients can create a challenging environment for nursing students. The high levels of distress contribute to a reduced cognitive function, impaired critical thinking skills, a decreased academic performance, and a reduced overall well-being [8]. Psychological distress also negatively influences nursing students’ motivation, commitment, and preservation in the nursing profession [9]. 

Studies have been found on the perceived psychological distress among nursing students. Depression refers to one’s lack of interest, involvement, and motivation; life satisfaction; and a sense of deprecation, worthlessness, and hopelessness [10]. The authors also defined anxiety as a sense of tension, fear, worry, or panic about a specific situation. Stress is characterized by nervous arousal, a sense of tension, irritability, and difficulty relaxing [11]. The prevalence rates for the reported anxiety and depression symptoms among nursing students were 41.9% and 43.1%, respectively, in the systematic review and meta-analysis that included 25 studies from various countries [4]; 60.6% and 42.5% in a study conducted in Spain [12]; 65.7% and 61.1% in a study implemented in Jordan [13,14]; and 57.8% and 45.9% in a study implemented in Singapore [15]. One study revealed that 31% of nursing students reported depression, 39% reported anxiety, and 42% reported stress [16]. 

Significant positive correlations were also found between psychological distress and academic stress, as well as between psychological distress and burnout [17]. For the context of this study, the Maslach Burnout Inventory has been used to measure three dimensions, which are emotional exhaustion, depersonalization, and personal accomplishment. Maslach and Jackson (1986) [18] defined emotional exhaustion as a sense of oneself being physically exhausted and emotionally drained by excessive academic demands and continuous educational stress. Depersonalization refers to impaired, distorted, and detached responses towards one’s academic pursuits and responsibilities [18]. The authors also stated that personal accomplishment refers to a sense of capability, competence, and achievement of one’s academic pursuits. One longitudinal study on psychological distress among first year nursing students found that depression, anxiety, and stress scores had increased significantly after a 4-year follow up [19]. The increases in stress and anxiety could be attributed to the greater academic and clinical stressors among nursing students as well as worrying about the future [19]. Hwang and Kim (2023) [20] found that there was a correlation between academic burnout, stress, anxiety, and depression. It was further found that students who perceived clinical experience stress had a higher level of academic burnout. Motta de Vasconcelos et al. (2018) [21] revealed that 14.29% experienced burnout and 10.98% had depressive symptoms, and they also found a correlation between burnout and depressive symptoms in ICU nurses.

Research also reported a high prevalence of depression among undergraduate nursing students in the Kingdom of Saudi Arabia, ranging between 43.3% and 31% among Saudi nursing students [22,23]. Students under the age of 23 and those that did not prefer the profession were more likely to experience greater depression than older students, or those who had a preference for nursing [23]. Young nursing students that suffered from depression lacked the coping skills of the older students, which may explain the difference in prevalence between students younger than 23 compared to those older than 23 [23]. Alsolais et al. (2021) [22] also found anxiety among Saudi nursing students to have a prevalence of 37.2%. It should be noted that this rate was within the context of the COVID-19 pandemic and, as such, it may reflect a higher rate than pre-pandemic. However, limited studies analyzed the relationship between psychological distress, academic stress, and burnout among Saudi nursing students. Continuous evaluation and addressing the psychological symptoms among nursing students, as well as examining their associated factors, is becoming paramount to ensure their mental well-being, promote academic success, and to foster optimal learning and professional development [24]. The aim of the present study was to determine the prevalence and association of psychological distress with academic stress and burnout among Saudi nursing students. 

The existing literature highlights a range of factors that contribute to psychological distress among undergraduate nursing students, one of which is the academic year in nursing education [25]. Research suggests that first-year students are more susceptible to stress and anxiety, primarily due to their lack of familiarity with the academic and social demands of college life [26]. Meanwhile, senior nursing students’ practice and social experiences enhance their adaptability and their strategies for coping with stress throughout their years of nursing education [27]. Additionally, the transition to college life introduces a new environment and higher academic expectations, which can be particularly overwhelming for these younger students [28]. Furthermore, research indicates that the dynamics of home life and family expectations are pivotal in shaping students’ experiences of stress and anxiety [29]. Pressures to succeed academically, combined with personal and financial responsibilities at home, significantly contribute to the psychological burden faced by students [30].

Psychological distress is a multidimensional mental health problem that mainly includes depression, anxiety, and stress. It can vary in intensity over time, ranging from common, normal feelings of vulnerability, sadness, and fear to severe issues that may become disabling [31]. These mental health challenges have intensified among nursing students, particularly during the COVID-19 pandemic [32]. The pandemic produced significant challenges in education, shifting traditional in-person classes to online formats, disrupting clinical placements, and increasing social isolation [33]. These abrupt changes heightened stress levels, anxiety, and uncertainty among nursing students [34]. 

Another factor contributing to the vulnerability to psychological distress is its neurobiological underpinnings [35]. Psychological distress, particularly related to depressive disorder, is characterized by impairments in cognition, emotional regulation, and neurovegetative symptoms. Its etiology involves several pathophysiological mechanisms, including altered neurotransmission, hypothalamic–pituitary–adrenal axis abnormalities, inflammation, reduced neuroplasticity, and network dysfunction [36]. The stressors induced by the pandemic, such as social isolation and disrupted routines, can lead to the hyperactivity of the HPA axis, causing elevated cortisol levels that affect the brain regions involved in emotion regulation, such as the prefrontal cortex and hippocampus [37]. This stress response is linked to decreased neurogenesis and neuroplasticity, further exacerbating psychological distress, especially depressive symptoms [38]. Additionally, chronic stress and inflammation can reduce levels of neurotransmitters like serotonin, norepinephrine, and dopamine, contributing to the neurobiological basis of depression [36].

## 2. Materials and Methods

### 2.1. Design, Sample, and Setting

This study used a cross-sectional, descriptive correlational design. A convenient sample of undergraduate nursing students was recruited from a nursing college in Riyadh, Kingdom of Saudi Arabia. The inclusion criteria comprised nursing students pursuing a Bachelor’s degree, who had proficiency in reading English, completion of at least one semester in the program and one clinical course, and voluntary consent to participate. Nursing students with a history of severe psychiatric disorders and those pursuing a Master’s degree or doctoral degree were excluded from this study. To determine the required sample size for statistical power, we conducted a power analysis using G* Power (version 3.1), considering 10 predictors, a 0.15 effect size, a 0.05 margin of error, and 95% statistical power. The results indicated that 172 student nurses were required for the multiple linear regression analysis.

### 2.2. Measurement

A demographic data form was developed and included age, gender, grade point average (GPA), study year, financial hardship, sleep problems, and physical and mental health issues. 

#### 2.2.1. Psychological Distress

To evaluate psychological distress, the DASS-21, which stands for Depression, Anxiety, and Stress Scale, was used [11]. The scale is widely used to measure the severity of symptoms of depression, anxiety, and stress. It consists of three subscales, each targeting a specific emotional state. The Depression subscale evaluates symptoms such as hopelessness, low self-esteem, and lack of interest, while the Anxiety subscale focuses on symptoms like restlessness, nervousness, and a sense of impending doom. The Stress subscale measures responses to various stressors, including difficulty relaxing and irritability. Participants rate each of the 21 items on a Likert-type scale, ranging from 0 = did not apply at all to 3 = applied very much or most of the time. Higher scores indicate higher depression, anxiety, and stress. The scores were interpreted using the manual for the DASS that was developed by [11]. The DASS scale is recognized as a reliable and valid instrument for evaluating emotional well-being and psychological distress. The internal consistency for each subscale ranged from 0.80 to 0.97 [39]. The Cronbach’s alpha coefficient for the DASS scale was 0.95 in this study.

#### 2.2.2. Academic Stress

Lin and Chen (2009) [40] developed the Academic Stress Inventory to gauge students’ academic stress levels. This scale comprises 34 questions addressing seven potential sources of stress related to academia including teachers’ stress, results stress, test stress, studying in group stress, peer stress, time-management stress, and self-inflicted stress. Participants rated all questions on a five-point Likert scale ranging from 1 = completely disagree to 5 = completely agree. Higher scores indicate elevated stress levels. The Cronbach reliability test and alpha values for possible sources of stress in this scale ranged from 0.85 to 0.92, and the overall alpha value for the entire scale was 0.90 [40]. The Cronbach’s alpha coefficient for the scale was 0.95 in this study. 

#### 2.2.3. Academic Burnout

The Maslach Burnout Inventory–Human Services Survey (MBI-HSS) was used to evaluate the academic burnout among the participants [41]. The survey was modified to better suit the students’ context, with work-related items replaced by items relevant to their studies [41]. In addition, the items related to patients were replaced with mentions of peers or others in general [41,42]. The scale consists of 22 items and employs a Likert-type scale ranging from 0 = never to 6 = every day. The participants were asked to rate their experiences across the following three dimensions: emotional exhaustion, depersonalization, and a lack of personal accomplishment. The range of the total score is between 0 and 132; a high score on the emotional exhaustion and depersonalization subscales, coupled with a low score on personal accomplishment, indicates the presence of burnout. The scores were interpreted using the Maslach Burnout Inventory Manual [43]. Previous research with university students demonstrated a good internal reliability for emotional exhaustion (α = 0.77), depersonalization (α = 0.80), and a lack of personal accomplishment (α = 0.68) [42]. The Cronbach’s alpha coefficient for the overall scale was 0.84 in this study.

### 2.3. Ethical Considerations

Prior to initiating data collection, this study was approved by the Institutional Review Board (IRB) at one of the educational institutions located in Riyadh, Saudi Arabia (Registration Number: 22-0494). Participants received thorough information regarding the study and its requirements, before providing consent. It was emphasized that participation in the study is voluntary, and data collection is carried out anonymously. Furthermore, participants were assured that their involvement carried no potential harm and would not impact their academic standing or progression in the college of nursing. They were also informed that their data would be treated with guaranteed confidentiality and security, strictly limited to research purposes. Participants were made aware of their right to refuse answering uncomfortable questions and to withdraw from the study at any time.

### 2.4. Data Collection 

Data for this study were collected through an online survey. We distributed a link to potential participants, enabling them to access the survey online. Participants who accessed the online survey link and agreed to participate were provided with written instructions for responding to the survey questions. The online survey link was accessible from February 2023 to October 2023, serving as the designated period for data collection.

### 2.5. Data Analysis

Data analyses were conducted using SPSS 29 ^®^ (SPSS Inc., Chicago, IL, USA). Descriptive statistics were used to describe the study variables. We performed reverse scoring for the BMI_HSS, as previously described [43], and calculated the total score using the summation method. We created three multiple regression models on the three subscales of the DASS (depression, anxiety, and stress), and one multiple regression model using the sum score from the DASS to measure psychological distress. The academic stress domains (teachers’ stress, results stress, test stress, studying in group stress, peer stress, time-management stress, and self-inflicted stress) and academic burnout domains (emotional exhaustion, depersonalization, and a lack of personal accomplishment) were used as predictors. All statistical assumptions, including multicollinearity and residual normality, for each regression model were thoroughly assessed and found to be met. A significance level of *p* < 0.05 was adopted for determining statistical significance. There were 246 participants who completed the survey. We excluded participants (n = 9) with missing data, resulting in data from 237 participants being utilized in the analyses.

## 3. Results

The study participants were predominantly female (93.2%), with an average age of 19.78 (SD = 1.43) and resided with their family (92.4%). A significant portion of participants were first-year students (35.9%), followed by second-year students (23.2%), third-year students (22.8%), and fourth-year students (18.1%). The average GPA was 3.94 (SD = 0.73). Most of the study participants reported no financial hardship (68.4%), no sleep disorders (53.2%), and no physical health problems (68.4%). 

Most of the participants reported no-to-mild depression (57.4%), 21.2% reported moderate depression, and 21.5% reported severe-to-extremely severe depression, with mean scores of 5.40 (SD = 2.73) for depression. Around 62.4% of the participants reported no-to-mild anxiety, 9.3% reported moderate anxiety, and 28.3% reported severe-to-extremely severe anxiety, with mean scores of 5.12 (SD = 2.3) for anxiety. The majority reported no-to-mild stress (79.7), 8.1% reported moderate stress, and 12.2% reported severe-to-extremely severe stress, with mean scores of 6.41 (SD = 2.08) for stress. The overall mean of academic stress among nursing students was 102.56 (SD = 28.18). Among the dimensions of academic stress, teachers’ stress had the highest mean score (29.09, SD = 8.08), while time-management stress was the lowest source of stress (9.17; SD = 3.58) for the participating nursing students. The participants reported moderate scores of teachers’ stress (56.1%), peer stress (51.9%), results stress (51.5%), studying in group stress (49.8%), and self-inflicted stress (47.7%), respectively. However, the participants’ scores were high in test stress (46%) and time-management stress (39.7%). 

The overall mean of burnout among the participating nursing students was 58.99 (SD = 21.60), with the highest mean pertaining to the ‘personal exhaustion’ dimension at 20.39 (SD = 9.76), whereas the lowest mean pertained to ‘depersonalization’, with a mean of 14.92 (SD = 5.85). Participants represent a moderate level of emotional exhaustion (43.5%), a high level of depersonalization (77.6%), and a low level of personal accomplishment (74.3%). 

The partial correlation matrix adjusting for age and gender indicates significant correlations among all study variables, except for depersonalization, which was not significantly correlated with results stress, test stress, studying in group stress, and self-inflicted stress (Table 1). 

The regression analysis showed studying in group stress, self-inflected stress, emotional exhaustion, depersonalization, and personal accomplishment to be significant predictors of the depression subscale. The regression model explains 50.6% of the variance in the depression subscale (Table 2). 

The regression analysis also showed studying in group stress, peer stress, and emotional exhaustion to be significant predictors of the anxiety subscale. The regression model explains 63.2% of the variance in the anxiety subscale (Table 2). 

Furthermore, studying in group stress, time-management stress, emotional exhaustion, and personal accomplishment were significant predictors of the stress subscale. The regression model explains 50.4% of the variance in the stress subscale (Table 3).

In the final regression analysis, where the DASS total score was used, studying in group stress, time-management stress, emotional exhaustion, depersonalization, and personal accomplishment were significant predictors of the overall psychological distress. The regression model explains 52.1% of the variance in the overall psychological distress (Table 4). 

## 4. Discussion

This study aimed to explore the prevalence of psychological distress and its association with academic stress and burnout among nursing students. The study found that 21.2% of the participants reported moderate depression and 21.5% reported severe-to-extremely severe depression, while 9.3% reported moderate anxiety and 28.3% reported severe-to-extremely severe anxiety. Only 8.1% reported moderate stress and 12.2% reported severe-to-extremely severe stress. The results also showed that stress related to studying in groups, time management, emotional exhaustion, depersonalization, and personal accomplishment were identified as significant predictors of psychological distress, explaining 52.1% of the psychological distress variance.

Our findings indicated that approximately one-third of the participants experienced some degree of psychological distress. One study found Saudi nursing students to have higher levels of depression, anxiety, loss of emotional control, and psychological distress compared to their Australian and South African peers [44]. Other studies reported a high prevalence of psychological distress among nursing students in Italy [27] and the United Kingdom [45]. However, one study reported a low prevalence of psychological distress among nursing students in the United States [34]. This inconsistency in the findings could be attributed to cultural and socioeconomic factors, the availability of leisure time, and variations in the student demographics being studied.

Factors such as cultural and socioeconomic factors and family and religious expectations could contribute to higher levels of psychological distress among Saudi nursing students [46,47]. Our study also found academic stress and burnout to be associated with psychological distress among Saudi nursing students. Specifically, the results demonstrated that studying in group stress, self-inflicted stress, emotional exhaustion, depersonalization, and personal accomplishment are significant predictors of nursing student depression. This can be attributed to several factors. Working with multiple individuals often entails managing conflicting schedules, communication challenges, and differences in learning styles [48], all of which may contribute to increased levels of depression among nursing students. Moreover, self-inflicted stress arises from internal pressures to excel academically, meet high standards, and fulfill personal expectations or career aspirations, leading students to impose unrealistic demands on themselves, ultimately increasing their levels of depression. These findings are consistent with a recent study that revealed exhaustion and depression as contributors to higher depression levels among college students [49]. Additionally, similar results have been reported among medical students, indicating that emotional exhaustion and depersonalization are both associated with their levels of depression [50].

Furthermore, the study findings reveal that group stress, peer stress, and emotional exhaustion are significant predictors of anxiety among undergraduate nursing students. Given that nursing students are highly prone to experiencing anxiety compared to students from other scientific fields [51], it becomes imperative to identify the determinants of anxiety among nursing students and introduce interventions to reduce it. These results serve this context by elucidating specific areas, where addressing them could decrease anxiety. These results are also consistent with those of [50], who demonstrated that emotional exhaustion is closely related to college students’ anxiety.

One of the novel findings in this study is that studying in group stress, time-management stress, emotional exhaustion, and personal accomplishment represent significant predictors of stress among undergraduate nursing students. These are significant findings that highlight the multifaceted nature of stress experienced by nursing students, encompassing factors related to group work dynamics, time management challenges, emotional resilience, and personal expectations. The identification of these predictors underscores the importance of addressing various aspects of student life to effectively manage stress levels. These results align with previous research indicating that time management is a key indicator of academic stress [52]. Additionally, our study showed that the presence of peers did not decrease stress levels [53]. Furthermore, these results are similar to those reported previously, which demonstrated that emotional exhaustion and a lack of personal accomplishment are major sources of stress for students [54].

The findings from this study also demonstrated that studying in group stress, time-management stress, emotional exhaustion, depersonalization, and personal accomplishment were significant predictors of overall psychological distress. These findings underscore that stressors stemming from group work dynamics, time-management challenges, and the dimensions of burnout, including emotional exhaustion, depersonalization, and personal accomplishment, represent significant sources of psychological distress among undergraduate nursing students. These findings emphasize the pressing need for interventions aimed at reducing stress related to group work, addressing time-management issues, and mitigating burnout. These findings are consistent with the concept of loss spirals elucidated in the Conservation of Resources theory [55], which suggests that individuals with limited resources are more susceptible to experiencing additional resource losses. In a detrimental cycle, initial resource deficits caused by stress and burnout can lead to subsequent losses of resources, such as psychological distress. Additionally, these findings align with a previous study that linked academic burnout dimensions to increased levels of psychological stress [56].

## 5. Implications of the Study

This research contributes to the current nursing literature focusing on academic stress, burnout, and psychological distress among nursing students. Additionally, it offers practical implications for nurse educators aiming to alleviate psychological distress among their undergraduate students. Our findings highlight that stressors arising from group work and time management are significant contributors to psychological distress in undergraduate nursing students. Consequently, nurse educators should implement interventions to mitigate these stressors stemming from group work and time management challenges. One strategy for achieving this is for nurse educators to establish cooperative zero-stress guidelines for students when engaging in group work. Additionally, introducing programs early in the semester that educate students on teamwork and effective time management can prove their benefits in reducing psychological distress. These proactive measures can empower students with the necessary skills and support to navigate stressors more effectively, fostering a conducive learning environment.

Furthermore, the study results revealed that three dimensions of burnout (emotional exhaustion, depersonalization, and reduced personal accomplishment) significantly contribute to psychological distress among undergraduate nursing students. Therefore, it is crucial to take actions aimed at alleviating burnout. Nurse educators could educate students about coping strategies. Monitoring could also be an effective method for the early detection of nursing students experiencing burnout, enabling timely intervention. Moreover, encouraging peer reporting could facilitate the identification of colleagues showing signs of burnout, allowing for appropriate support and assistance. By implementing these strategies, nurse educators can effectively mitigate psychological stress among their students.

This study recommends implementing formal mental health screening and support services tailored to the unique needs of nursing students. It also recommends fostering a culture that destigmatizes mental health and encourages help-seeking behavior among nursing students. Additionally, it recommends embedding mental health professionals into the nursing program to provide integrative and holistic team-based care, provide on-site counseling and support, and ensure seamless referrals for nursing students. It also recommends leveraging telehealth or mobile health applications for remote monitoring, to expand access to mental health services, identify trends, and inform personalized support interventions for nursing students.

## 6. Limitations and Future Research Directions

This study had several limitations. The cross-sectional design of the study facilitates the identification of associations rather than causal relationships between the studied variables. Further longitudinal and experimental research designs are suggested to establish these relationships. Moreover, the study was conducted among Saudi undergraduate nursing students, which may limit generalizability to other nursing students outside Saudi Arabia. Lastly, the data were gathered through self-report surveys, which might be susceptible to social desirability bias. Future investigations should aim to employ multiple sources to validate the study findings.

## 7. Conclusions

The study revealed that studying in group stress, time-management stress, emotional exhaustion, depersonalization, and personal accomplishment were significant predictors of overall psychological distress among undergraduate nursing students. Future research should consider these factors while designing strategies to decrease or prevent psychological distress among Saudi nursing students.

## Figures and Tables

**Table 1:** Correlation between the study variables (n = 237).

		Depression	Anxiety	Stress	Overall Psychological Distress	Teachers’ Stress	Results Stress	Test Stress	Studying in Group Stress	Peer Stress	Time-Management Stress	Self-Inflicted Stress	Overall Academic Stress Inventory	Emotional Exhaustion	Depersonalization	Personal Accomplishment	Overall Maslach Burnout Inventory
Depression	*r*																
	*p*																
Anxiety	*r*	0.780 *															
	*p*	<0.001 *															
Stress	*r*	0.848 *	0.815 *														
	*p*	<0.001 *	<0.001 *														
**Overall Psychological Distress**	*r*	0.939 *	0.917 *	0.952 *													
	*p*	<0.001 *	<0.001 *	<0.001 *													
Teachers’ stress	*r*	0.465 *	0.447 *	0.504 *	0.505 *												
	*p*	<0.001 *	<0.001 *	<0.001 *	<0.001 *												
Results stress	*r*	0.417 *	0.417 *	0.435 *	0.436 *	0.560 *											
	*p*	<0.001 *	<0.001 *	<0.001 *	<0.001 *	<0.001 *											
Test stress	*r*	0.427 *	0.427 *	0.472 *	0.499 *	0.677 *	0.651 *										
	*p*	<0.001 *	<0.001 *	<0.001 *	<0.001 *	<0.001 *	<0.001 *										
Studying in group stress	*r*	0.598 *	0.598 *	0.587 *	0.610 *	0.512 *	0.473 *	0.547 *									
	*p*	<0.001 *	<0.001 *	<0.001 *	<0.001 *	<0.001 *	<0.001 *	<0.001 *									
Peer stress	*r*	0.480 *	0.480 *	0.505 *	0.482 *	0.563 *	0.534 *	0.551 *	0.649 *								
	*p*	<0.001 *	<0.001 *	<0.001 *	<0.001 *	<0.001 *	<0.001 *	<0.001 *	<0.001 *								
Time-management stress	*r*	0.503 *	0.473 *	0.570 *	0.552 *	0.652 *	0.576 *	0.694 *	0.646 *	0.589 *							
	*p*	<0.001 *	<0.001 *	<0.001 *	<0.001 *	<0.001 *	<0.001 *	<0.001 *	<0.001 *	<0.001 *							
Self-inflicted stress	*r*	0.544 *	0.459 *	0.550 *	0.555 *	0.714 *	0.682 *	0.726 *	0.599 *	0.591 *	0.704 *						
	*p*	<0.001 *	<0.001 *	<0.001 *	<0.001 *	<0.001 *	<0.001 *	<0.001 *	<0.001 *	<0.001 *	<0.001 *						
**Overall Academic Stress Inventory**	*r*	0.596 *	0.544 *	0.634 *	0.633 *	0.780 *	0.776 *	0.843 *	0.762 *	0.764 *	0.832 *	0.873 *					
	*p*	<0.001 *	<0.001 *	<0.001 *	<0.001 *	<0.001 *	<0.001 *	<0.001 *	<0.001 *	<0.001 *	<0.001 *	<0.001 *					
Emotional exhaustion	*r*	0.507 *	0.470 *	0.522 *	0.535 *	0.345 *	0.334 *	0.370 *	0.425 *	0.388 *	0.375 *	0.433 *	0.503 *				
	*p*	<0.001 *	<0.001 *	<0.001 *	<0.001 *	<0.001 *	<0.001 *	<0.001 *	<0.001 *	<0.001 *	<0.001 *	<0.001 *	<0.001 *				
Depersonalization	*r*	0.142 *	0.195 *	0.195 *	0.213 *	0.110 *	0.110	0.106	0.125	0.136 *	0.131 *	0.115	0.162 *	0.617 *			
	*p*	0.030 **	0.004 *	0.001 *	0.001 *	0.007 *	0.091	0.105	0.057	0.037 **	0.044 **	0.079	0.013 **	<0.001 *			
Personal accomplishment	*r*	0.338 *	0.304 *	0.391 *	0.370 *	0.266 *	0.260 *	0.257 *	0.182 *	0.227 *	0.199 *	0.298 *	0.305 *	0.624 *	0.656 *		
	*p*	<0.001 *	<0.001 *	<0.001 *	<0.001 *	<0.001 *	<0.001 *	<0.001 *	0.005 *	0.001 *	0.002 *	<0.001 *	<0.001 *	<0.001 *	<0.001 *		
**Overall Maslach Burnout Inventory**	*r*	0.413 *	0.373 *	0.383 *	0.489 *	0.401 *	0.291 *	0.299 *	0.323 *	0.342 *	0.298 *	0.353 *	0.404 *	0.895 *	0.843 *	0.889 *	
	*p*	<0.001 *	<0.001 *	<0.001 *	<0.001 *	<0.001 *	<0.001 *	<0.001 *	<0.001 *	<0.001 *	<0.001 *	<0.001 *	<0.001 *	<0.001 *	<0.001 *	<0.001 *	

* Statistically significant at *p* ≤ 0.01. ** Statistically significant at *p* < 0.05.

**Table 2:** Multiple linear regression analysis showing the effect of different factors on depression (n = 237).

Variable	B	Beta	*t*	*p*	95% CI	
					LL	UL
**Academic Stress Inventory**						
Teachers’ stress	0.034	0.062	0.75	0.454	−0.056	0.125
Results stress	−0.026	−0.03	−0.396	0.692	−0.155	0.103
Test stress	0.12	0.122	1.434	0.153	−0.045	0.285
Studying in group stress	0.29	0.332	4.295	<0.001	0.157	0.424
Peer stress	−0.172	−0.148	−1.995	0.047	−0.342	−0.002
Time-management stress	0.157	0.125	1.48	0.14	−0.052	0.366
Self-inflicted stress	0.011	0.011	0.113	0.91	−0.186	0.209
**Maslach Burnout Inventory**						
Emotional exhaustion	0.122	0.265	3.225	0.001	0.047	0.196
Depersonalization	−0.077	−0.101	−1.33	0.185	−0.192	0.037
Personal accomplishment	0.053	0.108	1.428	0.155	−0.02	0.125

R^2^ = 0.632, F = 15.10, *p* < 0.001. F, *p*: f and *p* values for the model; R^2^: coefficient of determination; B: unstandardized coefficients; Beta: standardized coefficients; *t*: *t*-test of significance; LL: lower limit; UL: upper Limit.

**Table 3:** Multiple linear regression analysis showing the effect of different factors on stress (n = 237).

Variable	B	Beta	*t*	*p*	95% CI	
					LL	UL
**Academic Stress Inventory**						
Teachers’ Stress	0.015	0.023	0.309	0.757	−0.080	0.110
Results Stress	−0.039	−0.039	−0.568	0.570	−0.174	0.096
Test stress	−0.009	−0.008	−0.104	0.917	−0.183	0.164
Studying in group stress	0.272	0.269	3.829	0.000	0.132	0.412
Peer stress	0.039	0.029	0.434	0.665	−0.139	0.217
Time-management stress	0.327	0.226	2.934	0.004	0.107	0.547
Self-inflicted stress	0.109	0.088	1.032	0.303	−0.099	0.316
**Maslach Burnout Inventory**						
Emotional exhaustion	0.109	0.206	2.757	0.006	0.031	0.187
Depersonalization	−0.060	−0.068	−0.986	0.325	−0.180	0.060
Personal accomplishment	0.103	0.184	2.677	0.008	0.027	0.180

R^2^ = 0.504, F = 23.10, *p* < 0.001. F, *p*: f and *p* values for the model; R^2^: coefficient of determination; B: unstandardized coefficients; Beta: standardized coefficients; *t*: *t*-test of significance; LL: lower limit; UL: upper Limit.

**Table 4:** Multiple linear regression analysis showing the effect of different factors on the overall psychological distress (n = 237).

Variable	B	Beta	*t*	*p*	95% CI	
					LL	UL
**Academic Stress Inventory**						
Teachers’ Stress	0.056	0.033	0.441	0.660	−0.194	0.306
Results Stress	−0.072	−0.027	−0.401	0.689	−0.428	0.283
Test stress	−0.051	−0.017	−0.219	0.827	−0.507	0.405
Studying in group stress	0.908	0.336	4.867	<0.001	0.540	1.276
Peer stress	−0.127	−0.035	−0.534	0.594	−0.596	0.341
Time-management stress	0.663	0.171	2.263	0.025	0.086	1.241
Self-inflicted stress	0.328	0.100	1.186	0.237	−0.217	0.873
**Maslach Burnout Inventory**						
Emotional exhaustion	0.396	0.279	3.802	<0.001	0.191	0.601
Depersonalization	−0.356	−0.151	−2.224	0.027	−0.672	−0.041
Personal accomplishment	0.265	0.176	2.612	0.010	0.065	0.465

R^2^ = 0.521, F = 24.57, *p* < 0.001. F, *p*: f and *p* values for the model; R^2^: coefficient of determination; B: unstandardized coefficients; Beta: standardized coefficients; *t*: *t*-test of significance; LL: lower limit; UL: upper limit.

## Data Availability

The datasets generated and/or analyzed during the current study are not publicly available due to data privacy, but are available from the corresponding author upon reasonable request.

## Abbreviations

DASS: Depression, Anxiety, and Stress Scale.
